# Prevalence and clinical correlates of insomnia symptoms and their association with quality of life in Chinese patients with HBV-related liver disease

**DOI:** 10.7717/peerj.10956

**Published:** 2021-03-04

**Authors:** Jing Zhao, Mei Liu, Gabor S. Ungvari, Chee H. Ng, Ines Hang Iao Chow, Ting Wang, Yu Chen, Zhongping Duan, Yu-Tao Xiang, Su-Jun Zheng

**Affiliations:** 1 Beijing YouAn Hospital, Capital Medical University, Beijing, China; 2 University of Notre Dame Australia, Fremantle, Australia; 3 Division of Psychiatry, School of Medicine, University of Western Australia / Graylands Hospital, Perth, Australia; 4 Department of Psychiatry, The Melbourne Clinic and St Vincent’s Hospital, University of Melbourne, Richmond, Victoria, Australia; 5 Center for Cognition and Brain Sciences, University of Macau, Macao SAR, China; 6 Institute of Advanced Studies in Humanities and Social Sciences, University of Macau, Macao SAR, China

**Keywords:** HBV-related liver disease, Insomnia, China

## Abstract

**Objective:**

This study aimed to describe the one-month prevalence of insomnia symptoms (insomnia hereafter) and the demographic and clinical correlates, and its association with quality of life (QOL) in Chinese patients with HBV-related liver disease.

**Method:**

A total of 689 patients with HBV-related liver disease in Beijing, China formed the study sample. Three forms of insomnia including difficulty initiating sleep (DIS), difficulty maintaining sleep (DMS) and early morning awakening (EMA) were assessed using standardized questions. QOL was measured using the Medical Outcomes Study Short Form 12 (SF-12).

**Results:**

The one-month prevalence of at least one type of insomnia was 69.5%, while DIS, DMS and EMA were 60.4%, 54.7% and 50.9%, respectively. Only 4.8% of patients suffering from insomnia received treatment. Multiple logistic regression analyses revealed that pre-existing medical conditions were positively associated with DIS and EMA; patients with more severe depressive symptoms were more likely to have DIS, DMS and EMA; local residents were less likely to have DIS; and those who were married and older were more likely to have DMS. Insomnia was not independently associated with QOL.

**Conclusions:**

Insomnia is common in Chinese patients with HBV-related liver disease with a very low rate of treatment. Greater attention should be given to identify and treat insomnia in this patient population.

## Introduction

Hepatitis B virus (HBV) infection is a common infectious disease with around 350–400 million HBV carriers worldwide (Custer et al., 2004). In China, there are approximately 93 million HBV carriers and among them, 30 million suffer from chronic hepatitis B (Liang et al., 2009). HBV infection is progressive and may lead to cirrhosis, liver failure and hepatocellular carcinoma (HCC), causing significant morbidity and mortality as well as considerable socioeconomic burden ( Polaris Observatory Collaborators, 2016). It is estimated that the annual direct and indirect health cost for a patient suffering from compensated or decompensated liver cirrhosis is USD 5,100 and USD 5,200, respectively in China (Lu & Zhuang, 2009).

In recent years psychiatric comorbidities in HBV-infection have gained increasing attention. Evidence suggests that comorbid psychiatric disorders are often underestimated (Duan et al., 2012; Gutteling et al., 2010). In a study involving 40 patients each with HBV-related acute-on-chronic liver failure (ACLF), HBV-related cirrhosis or chronic hepatitis B (CHB), patients with ACLF and cirrhosis reported more severe depressive symptoms with the mean Hamilton Rating Scale for Depression (HAMD) total score of 11.58 (SD=9.48) and 10.60 (SD=7.51), compared to CHB patients and healthy control with the mean HAMD total score of 6.03 (SD=6.91) and 5.30 (SD=5.51), respectively (Duan et al., 2012).

Insomnia symptoms (insomnia hereafter) are common health challenges in both the general population and those with major medical conditions due to their considerable impact on quality of life (QOL), work absenteeism, and societal cost (Burman, 2017; Stewart et al., 2014). In addition, insomnia is associated with an increased risk for hypertension, type 2 diabetes and certain types of cancers (Liang et al., 2012; Jarrin et al., 2018; Huang et al., 2015).

To the best of our knowledge, there was only one study that examined the prevalence of insomnia in HBV-infected patients (Guo et al., 2017). The study measured early, middle and late insomnia using items 4, 5 and 6 of the HAMD in 120 patients with HBV-related diseases and 40 matched healthy controls. The frequencies of early, middle, late and any type of insomnia were 39.2%, 42.5%, 48.3% and 64.2%, respectively, compared to 22.5%, 10.0%, 25.0% and 35.0% in healthy controls. The small sample size and lack of standardized measures on insomnia however limited the generalizability of these findings. QOL is a comprehensive health outcome widely used in clinical practice and research. Although previous studies found that insomnia has a negative association with QOL (Olfson et al., 2018; Khan & Aouad, 2017), this has not been examined specifically in patients with HBV infection.

Hence the aim of this study was to examine the prevalence of insomnia in the four clinical stages of chronic HBV infection (HBV carrier, CHB, hepatitis B cirrhosis, and HBV-related HCC) and the demographic and clinical correlates, and its association with QOL.

## Methods

### Study setting and participants

This study was conducted between September 1, 2014 and January 31, 2015 in Beijing YouAn Hospital that is a major infectious hospital in China and also a university-affiliated teaching hospital with 800 beds serving patients nationwide. Inpatients and outpatients were consecutively recruited if they were (1) aged 18 years or above; (2) diagnosed as HBV carrier, CHB, hepatitis B cirrhosis or HCC according to the Guidelines of Prevention and Treatment for Chronic Hepatitis B (2010 version) (Jia & Li, 2011) and the Recommendations of the Asian Pacific Association for the Study of the Liver (APASL) for the management of hepatocellular carcinoma (Sarin et al., 2009); (3) Chinese descent; (4) able to communicate adequately, tolerate a one-hour interview and comprehend the purpose of the study. The study protocol was approved by the Beijing YouAn Hospital Clinical Research Ethics committee. All patients provided written informed consent.

### Data collection

Each patient was interviewed face-to-face by one of two trained physicians with at least three-year clinical and research experience. Socio-demographic data such as age, gender, education, residence, marital status, personal monthly income, public health insurance were collected using a form designed for this study. Clinical data such as family history of psychiatric disorders, current use of alcohol, diagnoses of HBV-related liver disease, pre-existing medical conditions, age of onset of HBV, duration of HBV-related liver disease, number of hospitalizations were collected from a review of the medical records.

QOL was measured with the Chinese version of the Medical Outcomes Study Short Form 12 (SF-12) (Zhang et al., 2011; Perruccio et al., 2020). The SF-12 is a generic instrument with 12 items addressing eight health domains: physical functioning, role limitations due to physical problems, bodily pain, vitality, and social functioning as well as role limitations each related to emotional problems and mental health. For the purpose of statistical analysis, the first four domains were collapsed into a physical component score, while the remaining four domains formed a mental health component score. A higher score on SF-12 indicates better QOL. Global Assessment of Functioning (GAF) was used to evaluate overall psychosocial and occupational functioning. The total scale ranges from 1 to 100, with a higher score indicating more daily activities (Aas, Sonesson & Torp, 2018). The 10-item Montgomery-Asberg Scale–Chinese version (C-MADRS) was used to measure the severity of depressive symptoms within the past week (Liu et al., 2014; Zhong et al., 2011). A higher score indicates a worse condition. An alcohol user was defined as one who drank at least one alcoholic beverage each month in the last year (Xiang et al., 2009).

The presence of three basic forms of insomnia during the past month was examined by asking three standardized questions: “*Do you have difficulties in falling asleep?”* for difficulty initiating sleep (DIS); “*Do you have difficulties in maintaining sleep and wake up often?*” for difficulty maintaining sleep (DMS); and for early morning awakening (EMA) *“Do you wake up in the midnight or early morning and then have difficulties in* falling asleep again?” Following previous studies (Zhao et al., 2019; Liu & Zhou, 2002; Chiu et al., 1999), patients were considered “having insomnia” in the case that they answered “often” to anyone of the three questions. Furthermore, treatment for insomnia in the past month was also asked.

### Statistical analysis

Data were analyzed using SPSS 21.0 for Windows. Comparisons between insomnia and non-insomnia groups in terms of demographic and clinical variables were performed by chi-square tests, t-tests and Mann–Whitney U test, as appropriate. QOL was compared between insomnia and non-insomnia groups using analysis of covariance (ANCOVA) after controlling for the potentially confounding effects of variables that significantly differed between the two groups in univariate analyses. The independent associations between demographic and clinical characteristics and each type of insomnia were conducted by multiple logistic regression analyses with the “Enter” method. Each type of insomnia was entered as the dependent variable separately, while the demographic and clinical characteristics including treatment status (in-or outpatient), age, sex, marital status, residence, living alone, education, personal monthly income, health insurance, family history of psychiatric disorders, current use of alcohol, diagnoses of HBV-related liver disease, pre-existing medical conditions, age of onset of HBV, duration of HBV-related liver disease, number of hospitalizations, MADRS and GAF scores were entered as the independent variables. The level of significance was set at 0.05 (two-tailed).

## Results

**Figure 1:**
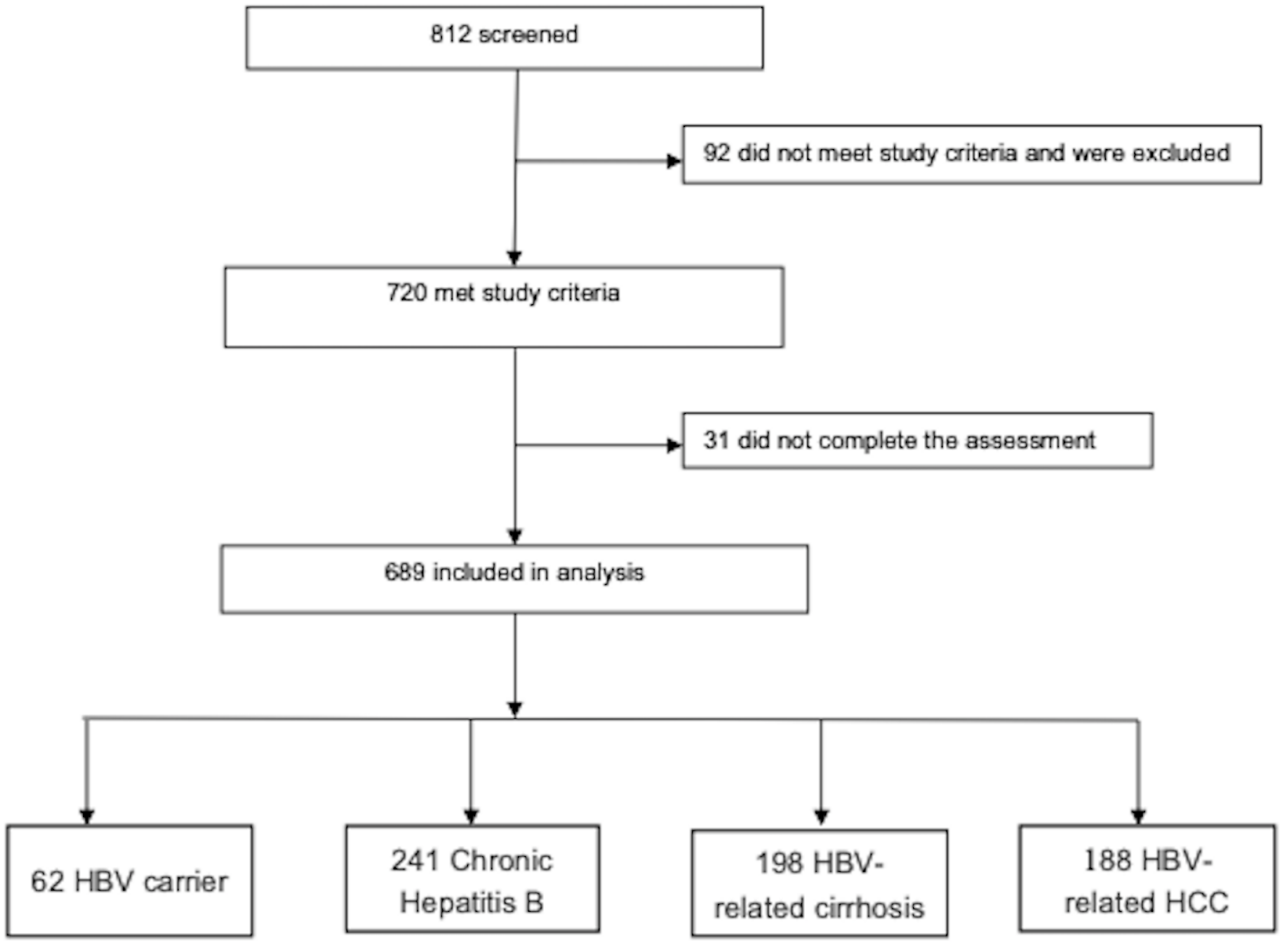
Flow diagram of the recruitment.

As shown in Fig. 1, altogether 812 patients with chronic HBV infection were invited to participate in the study of which 720 met study entry criteria resulting in a participation rate of 88.7%. However, only 689 completed the assessment and were included in the analyses. The prevalence of DIS, DMS, EMA and at least one type of insomnia was 60.4%, 54.7%, 50.9% and 69.5%, respectively. The proportions of patients reporting one, two and three types of insomnia were 15.2%, 12.0% and 42.2%, respectively. Only 4.8% of patients with insomnia reported taking “sleep-enhancing drugs”.

**Table 1:** Basic demographic and clinical characteristics of the sample.

	HBV carrier (*n* = 62)		Chronic Hepatitis B (*n* = 241)		HBV-related cirrhosis (*n* = 198)		HBV-related HCC (*n* = 188)	
	*N*	%	*N*	%	*N*	%	*N*	%
Inpatients	0	0	0	0	198	100.0	188	100.0
Male sex	34	54.8	157	65.1	153	77.3	152	80.9
Married	48	77.4	198	82.2	181	91.4	172	91.5
Local residents	27	43.5	81	33.6	84	42.4	100	53.2
Living alone	2	3.2	6	2.5	9	4.5	5	2.7
Personal income <3000 yuan	15	24.2	55	22.8	78	39.4	80	42.6
Having health insurance	62	100.0	237	98.3	191	96.5	185	98.4
Family history of psychiatric disorders	2	3.2	8	3.3	2	1.0	5	2.7
Current alcohol use	15	24.2	47	19.5	34	17.2	28	14.9
Pre-existing conditions	21	33.9	77	32.0	93	47.0	112	59.6
DIS	34	54.8	127	52.7	129	65.2	126	67.0
DMS	29	46.8	109	45.2	113	57.1	126	67.0
EMA	27	43.5	97	40.2	110	55.6	117	62.2
Insomnia symptoms	43	69.4	151	62.7	145	73.2	140	74.5
	Mean	SD	Mean	SD	Mean	SD	Mean	SD
Age (years)	37.1	11.9	39.4	12.2	51.9	11.2	58.2	8.6
Education (years)	11.7	4.1	11.5	3.8	10.8	5.8	11.1	4.9
Age of onset of HBV (years)	26.9	14.8	28.9	12.8	37.0	14.3	40.0	13.6
Duration of HBV-related liver disease (years)	11.5	9.0	10.8	9.5	15.1	12.5	17.9	11.8
Number of hospitalizations	0.1	0.3	0.4	0.6	2.4	3.9	4.2	3.5
MADRS total	6.0	8.6	5.4	6.8	6.9	7.6	8.4	7.9
GAF total	76.0	13.6	75.3	13.6	74.3	13.2	71.8	12.8

**Notes.**

DIS Difficulty initiating sleep DMS Difficulty maintaining sleep EMA Early morning awakening GAF Global Assessment of Functioning HCC Hepatocellular Carcinoma MADRS Montgomery-Asberg Depression Scale

Table 1 shows the basic demographic and clinical characteristics of participants by HBV-related liver diseases. The prevalence estimates of insomnia by sex and age are shown in Table 2. The basic demographic and clinical data were compared between insomnia and non-insomnia groups (Table 3). Patients with insomnia were more likely to be inpatients, have pre-existing medical conditions, cirrhosis and HCC, more hospitalizations and had more severe depressive symptoms and poorer psychosocial functioning. After controlling for the above variables that were significantly different between the two groups, no significant difference was found in the physical (*F*_(7,668)_ = 0.1, *P* = 0.75) and mental (*F*_(7,668)_ = 1.3, *P* = 0.24) domain of QOL.

**Table 2:** Prevalence of insomnia by age and sex.

Age (years)	DIS (*n* = 416)			DMS (*n* = 377)			EMA (*n* = 351)		
	Female % (95% CI.)	Male % (95% CI.)	Total % (95% CI.)	Female % (95% CI.)	Male % (95% CI.)	Total % (95% CI.)	Female % (95% CI.)	Male % (95% CI.)	Total % (95% CI.)
<50	65.3	54.6	57.5	56.8	47.0	49.7	43.2	46.2	45.4
	(0.56–0.75)	(0.48–0.61)	(0.52–0.63)	(0.47–0.67)	(0.41–0.53)	(0.44–0.55)	(0.33–0.53)	(0.40–0.52)	(0.40–0.51)
≥50	59.2	64.9	63.3	57.1	60.8	59.8	54.1	57.6	56.6
	(0.49–0.69)	(0.59–0.71)	(0.58–0.68)	(0.47–0.67)	(0.55–0.67)	(0.55–0.65)	(0.44–0.64)	(0.51–0.64)	(0.51–0.62)
Total	62.2	59.7	60.4	57.0	53.8	54.7	48.7	51.8	50.9
	(0.55–0.69)	(0.55–0.64)	(0.57–0.64)	(0.50–0.64)	(0.49–0.58)	(0.51–0.58)	(0.42–0.56)	(0.47–0.56)	(0.47–0.55)

**Notes.**

DIS Difficulty initiating sleep DMS Difficulty maintaining sleep EMA Early morning awakening

**Table 3:** Comparison between patients with and without insomnia symptoms with respect to basic demographic and clinical characteristics.

	Total sample (*n* = 689)		Insomnia symptoms (*n* = 479)		No insomnia symptoms (*n* = 210)		Statistics	
	N	%	N	%	N	%	*χ*^2^	p
Inpatients	386	56.0	285	59.5	101	48.1	7.7	**0.006**
Male sex	496	72.0	344	71.8	152	72.4	0.02	0.88
Married	599	86.9	419	87.5	180	85.7	0.40	0.53
Local residents	292	42.4	201	42.0	91	43.3	0.11	0.74
Living alone	22	3.2	16	3.3	6	2.9	0.11	0.74
Personal income <3000 yuan	228	33.1	157	32.8	71	33.8	0.07	0.79
Having health insurance	675	98.0	469	97.9	206	98.1	0.03	0.88
Family history of psychiatric disorders	17	2.5	10	2.1	7	3.3	0.94	0.33
Current drinker	124	18.0	90	18.8	34	16.2	0.67	0.41
Pre-existing conditions	303	44.0	229	47.8	74	35.2	9.36	**0.002**
Diagnose of HBV-related liver disease							8.82	**0.03**
Carrier	62	9.0	43	9.0	19	9.0		
Hepatitis	241	35.0	151	31.5	90	42.9		
Cirrhosis	198	28.7	145	30.3	53	25.2		
HCC	188	27.3	140	29.2	48	22.9		
	Mean	SD	Mean	SD	Mean	SD	T / Z	p
Age (years)	48.0	13.8	48.5	13.5	46.6	14.4	1.53	0.13
Education (years)	11.2	4.8	11.1	4.1	11.4	6.0	−0.20	0.84
Age of onset of HBV (years)	34.1	14.6	34.1	14.3	34.0	15.1	0.26	0.80
Duration of HBV-related liver disease (years)	14.0	11.4	14.5	11.4	13.0	11.3	1.58	0.12
Number of hospitalizations	2.0	3.2	2.2	3.5	1.5	2.7	3.32	**0.001**
MADRS total	6.7	7.6	8.8	8.0	2.0	3.2	13.79	**<0.001**
GAF total	74.2	13.3	70.4	12.9	82.6	10.1	−11.41	**<0.001**
SF-12 physical	65.2	13.9	66.2	14.6	62.9	12.0	4.00	**<0.001**
SF-12 mental	53.9	14.9	53.3	15.6	55.2	13.1	−1.61	0.11

**Notes.**

Bolded values are *p* < 0.05.

GAF Global Assessment of Functioning HCC Hepatocellular Carcinoma MADRS Montgomery-Asberg Depression Scale SF-12 Medical Outcomes Study Short Form 12

Multiple logistic regression analyses revealed that pre-existing medical conditions were positively associated with DIS and EMA and patients with more severe depressive symptoms were more likely to have DIS, DMS and EMA. Local residents were less likely to have DIS and married and older patients were more likely to have DMS (Table 4).

**Table 4:** Socio-demographic correlates of insomnia symptoms (logistic regression analysis).

	DIS			DMS			EMA		
	p	OR	95% CI	p	OR	95% CI	p	OR	95% CI
Inpatients	0.77	0.8	0.3–2.0	0.89	1.0	0.4–2.4	0.65	0.8	0.3–1.9
Male sex	0.21	0.7	0.5–1.2	0.26	0.8	0.5–1.2	0.54	1.2	0.7–1.8
Married	0.37	1.3	0.7–2.5	**0.04**	1.9	1.04–3.5	0.16	1.6	0.8–2.9
Local residents	**0.03**	0.6	0.4–0.96	0.15	0.8	0.5–1.1	0.96	1.0	0.7–1.5
Living alone	0.64	1.4	0.4–4.8	0.64	1.3	0.4–4.4	0.996	1.0	0.3–3.4
Personal income <3000 yuan	0.10	0.7	0.4–1.1	0.64	0.9	0.6–1.4	0.49	0.9	0.6–1.3
Having health insurance	0.18	0.4	0.1–1.6	0.62	0.7	0.2–2.6	0.42	0.6	0.2–2.2
Family history of psychiatric disorders	0.07	0.3	0.1–1.1	0.19	0.4	0.1–1.5	0.20	0.5	0.1–1.5
Current drinker	0.41	1.2	0.7–2.0	0.16	1.4	0.9–2.3	0.48	1.2	0.7–1.9
Pre-existing conditions	**0.01**	1.8	1.2–2.6	0.23	1.3	0.9–1.9	**0.01**	1.7	1.1–2.5
Diagnose of HBV-related liver disease									
Carrier		1.0			1.0			1.0	
Hepatitis	0.62	0.8	0.4–1.7	0.61	0.8	0.4–1.6	0.40	0.8	0.4–1.5
Cirrhosis	0.70	1.2	0.5–2.5	0.74	0.9	0.4–1.9	0.78	0.9	0.4–1.9
HCC	0.77	0.9	0.4–2.1	0.89	1.1	0.5–2.4	0.66	0.8	0.4–1.9
Age (years)	0.17	1.0	0.99–1.1	**0.03**	1.1	1.01–1.1	0.20	1.0	0.99–1.1
Education (years)	0.33	1.0	0.9–1.02	0.54	1.0	0.9–1.03	0.57	1.0	0.9–1.03
Age of onset of HBV (years)	0.11	1.0	0.9–1.01	0.09	1.0	0.9–1.01	0.55	1.0	0.9–1.03
Duration of HBV-related liver disease (years)	0.46	1.0	0.9–1.03	0.24	1.0	0.9–1.02	0.46	1.0	0.9–1.03
Number of hospitalizations	0.69	1.0	0.9–1.1	0.60	1.0	0.9–1.1	0.41	1.0	0.96–1.1
MADRS total	**<0.001**	1.3	1.2–1.4	**<0.001**	1.2	1.2–1.3	**<0.001**	1.2	1.2–1.3
GAF total	0.23	1.0	0.97–1.01	0.48	1.0	0.97–1.01	0.26	1.0	0.97–1.01

**Notes.**

Bolded values are *p* < 0.05.

DIS Difficulty initiating sleep DMS Difficulty maintaining sleep EMA Early morning awakening GAF Global Assessment of Functioning HCC Hepatocellular Carcinoma MADRS Montgomery- Asberg Depression Scale

## Discussion

To the best of our knowledge, this was the first study that examined insomnia in patients with chronic HBV infection using standardized questions. The definitions and measurements of the different types of insomnia varied from study to study (Chiu et al., 2012). This investigation focused on three basic forms of insomnia, i.e., DIS, DMS and EMA (Ohayon, 2002; Xiang et al., 2008). The prevalence of any type of insomnia (69.5%) found in this study is consistent with the rate (64.2%) reported in Chinese patients with HBV-related diseases (Guo et al., 2017) but slightly higher than the pooled findings (approximately 50%) in other chronic conditions including diabetes, hypertension, congestive heart failure or depression (Katz & McHorney, 1998). Furthermore, the prevalence rates of DIS, DMS, EMA and at least one type of insomnia in patients with chronic HBV infections in this study were significantly higher than the corresponding rates of 7.0%, 8.0%, 4.9%, and 9.2% in the Chinese general population, respectively (Xiang et al., 2008). Due to the inconsistent definitions of insomnia, different insomnia timeframe (e.g., current and past month) as well as different sampling methods (convenience vs. consecutive) (Ohayon, 2002; Franzen et al., 2010), comparisons of results across studies should be made with caution.

The high frequency of insomnia in HBV-related diseases may be accounted for by several factors including antiviral medications, disease-related physical symptoms and anxiety resulting from high treatment costs, lack of physical activity, less time spent outdoors and daytime sleeping due to fatigue and increased inflammatory cytokines (Guo et al., 2017; Irwin, 2001). It is not surprising that there is a greater likelihood of insomnia in patients with cirrhosis and HCC.

Due to the negative influence of HBV-related diseases and related problems, insomnia was expected to be associated with poor QOL. However, after controlling for demographic and clinical variables, no significant difference between the non- and insomnia groups in both physical and mental domains of QOL was found. We assume that some factors, such as psychiatric and physical comorbidity and impaired functioning might moderate the relationships between insomnia and QOL. Moreover, the SF-12 is a generic, rather than a disease-specific measure on QOL and perhaps is not sensitive enough to detect minor changes in QOL.

Only 4.8% of the patients with insomnia in this study reported taking “sleep-enhancing drugs”. Reasons for the low treatment rate in this population may be due to the traditional Chinese view of insomnia as not being a medical condition (Singh & Zhao, 2017). In addition, there may be inadequate assessment and treatment of insomnia in Chinese infectious disease hospitals owing to a lack of awareness, knowledge and skills to manage such conditions. Moreover, insomnia symptoms, rather than a clinical diagnosis of insomnia, were assessed in this study. Compared to clinical insomnia, insomnia symptoms are less likely to cause negative health outcomes, which may explain the low treatment rate.

Similar to earlier findings (Guo et al., 2017; Franzen et al., 2010; Patel, Steinberg & Patel, 2018), advanced age and depressive symptoms were associated with insomnia. It should be noted that due to its cross-sectional design, this study could not establish the causality and directionality between depressive symptoms and insomnia. Further, MADRS also includes insomnia items, which may lead to over-estimation of the association between depression and insomnia in this study. Married status was associated with insomnia, which is consistent with the results of previous surveys in the Chinese general population (Xiang et al., 2008). In today’s fast-changing Chinese society, HBV-related discrimination, increasingly complex personal relationships, social stress, the gradual dissolution of the traditional family structure are factors that may lead to marriage-related problems resulting in the higher likelihood of insomnia (Yang & Wu, 2011). Consistent with Western and Chinese studies (Xiang et al., 2008; Ohayon & Lemoine, 2002), insomnia was associated with pre-existing medical conditions in this study. Around two-thirds of patients were not local residents. As this hospital accepts patients nationwide, due to the health insurance policy, most treatment costs of non-residents were not reimbursed by their health insurance. The high burden of treatment costs to the patients is often a significant trigger for anxiety and secondary insomnia.

Several methodological limitations should be mentioned in this study. First, this is a cross-sectional study; therefore, the causal relationship between insomnia and socio-demographic and clinical variables could not be examined. Second, only patients with chronic HBV infection from one major hospital were included, thus the results may not be generalized to the whole country. Additionally, as all participants were hospital-based, this could lead to a selection bias and overestimation of the rates of insomnia. Third, insomnia was evaluated by self-reported standardized questions, thus recall bias could not be excluded. Although the three standardized questions on insomnia symptoms have been widely used previously (Zhao et al., 2019; Liu & Zhou, 2002; Chiu et al., 1999), their psychometric properties have not been examined in Chinese patients with HBV-related liver disease. In addition, the possibility of potential overlap between DMS and EMA could not be excluded. Fourth, some factors related to insomnia and its treatment, such as anxiety and social support, were not examined. Finally, apart from sleep-enhancing drugs, certain psychosocial interventions, such as cognitive behavioural therapy (CBT), were also used for insomnia (Haynes et al., 2018; Asarnow & Manber, 2019). However, use of such psychosocial interventions for insomnia was not examined.

## Conclusions

In conclusion, given the adverse consequences of insomnia coupled with its high prevalence found in this survey, attempts should be made to identify and treat insomnia in patients with HBV-related diseases.

##  Supplemental Information

10.7717/peerj.10956/supp-1 Supplemental Information 1 Raw data

## Additional Information and Declarations

### Data Availability

The following information was supplied regarding data availability: Raw data are available as a Supplemental File.
